# Supplementary material for: MetaRibo-Seq measures translation in microbiomes
Source: Nat Commun. 2020 Jun 29;11:3268. doi: 10.1038/s41467-020-17081-z (PMC7324362; doi:10.1038/s41467-020-17081-z)
Supplement: Supplementary file 10 — Supplementary Data 7 [file 41467_2020_17081_MOESM10_ESM.zip › File2/Confidence_High_Taxonomy/223032_out.krona.html]

Javascript must be enabled to view this page.

members
magnitude
magnitudeUnassigned
count
unassigned
taxon
rank

223032\_out

28

SRS013965\_contig\_number\_41232
1

24
2
superkingdom

1239
23
3
phylum

SRS014235\_contig\_number\_49779SRS014736\_contig\_number\_2284SRS018984\_contig\_number\_11859

class
186801
19

19
order
186802

family
216572
7

459786
genus
7

1945593
species

SRS015431\_contig\_number\_88165SRS020869\_contig\_number\_34021SRS042628\_contig\_number\_37902SRS098073\_contig\_number\_17468SRS1041031\_contig\_number\_29765SRS1041033\_contig\_number\_30133SRS104400\_contig\_number\_contig-100\_38.333890
7

1897048
species

SRS019068\_contig\_number\_25219SRS019445\_contig\_number\_15428SRS049446\_contig\_number\_14548SRS049773\_contig\_number\_34602SRS064276\_contig\_number\_42153SRS144362\_contig\_number\_30523SRS147346\_contig\_number\_47725SRS149325\_contig\_number\_16253
8

family
186803
4

4

SRS014412\_contig\_number\_14047SRS015890\_contig\_number\_15313SRS024132\_contig\_number\_47398SRS148159\_contig\_number\_37419
species
1951976

1

SRS1041140\_contig\_number\_18855
species
1897037

phylum
976
1

117743
class
1

1
order
200644

1
family
49546

1
363408
genus

2058134
species

SRS148196\_contig\_number\_38014
1


SRS064757\_contig\_number\_contig-100\_14109.66555SRS104975\_contig\_number\_17969SRS143895\_contig\_number\_37260
3
